# Supplementary material for: Enhanced geocoding precision for location inference of tweet text using spaCy, Nominatim and Google Maps. A comparative analysis of the influence of data selection
Source: PLoS One. 2023 Mar 15;18(3):e0282942. doi: 10.1371/journal.pone.0282942 (PMC10016707; doi:10.1371/journal.pone.0282942)
Supplement: S1 File — (PDF) [file pone.0282942.s001.pdf]

ResearchGate

See discussions, stats, and author profiles for this publication at: https://www.researchgate.net/publication/358405020

Serere et al Extracting and Geocoding Locations in Social Media Posts: A Comparative Analysis

Conference Paper · February 2022

CITATIONS
0

READS
36

4 authors, including:

Helen Ngonidzashe Serere
University of Salzburg
1 PUBLICATION **0** CITATIONS
SEE PROFILE

Bernd Resch
University of Salzburg
170 PUBLICATIONS **3,069** CITATIONS
SEE PROFILE

Clemens Havas
Fachhochschule Salzburg
18 PUBLICATIONS **308** CITATIONS
SEE PROFILE

Some of the authors of this publication are also working on these related projects:

Project E2mC Horizon2020 View project

Project Extarcting and Geocoding inferred Tweet locations View project

All content following this page was uploaded by Helen Ngonidzashe Serere on 07 February 2022.

The user has requested enhancement of the downloaded file.

GI_Forum 2021, Issue 2
Page: 167 - 173
Short Paper
Corresponding Author:
helenngonidzashe.serere@plus.ac.at
DOI: 10.1553/giscience2021_02_s167

Extracting and Geocoding Locations in Social Media Posts: A Comparative Analysis

Helen Ngonidzashe Serere, Bernd Resch, Clemens Rudolf Havas and Andreas Petutschnig

University of Salzburg, Austria

Abstract

Geo-social media have become an established data source for spatial analysis of geographic and social processes in various fields. However, only a small share of geo-social media data are explicitly georeferenced, which often compromises the reliability of the analysis results by excluding large volumes of data from the analysis. To increase the number of georeferenced tweets, inferred locations can be extracted from the texts of social media posts. We propose a customized workflow for location extraction from tweets and subsequent geocoding. We compare the results of two methods: DBpedia Spotlight (using linked Wikipedia entities), and spaCy combined with the geocoding methods of OpenStreetMap Nominatim. The results suggest that the workflow using spaCy and Nominatim identifies more locations than DBpedia Spotlight. For 50,616 tweets posted within California, USA, the granularity of the extracted locations is reasonable. However, several directions for future research were identified, including improved semantic analysis, the creation of a cascading workflow, and the need to integrate different data sources in order to increase reliability and spatial accuracy.

Keywords:

location extraction, geocoding, Twitter, DBpedia Spotlight, spaCy

1 Introduction

'Geo-social media data' refers to social media posts that have a geospatial reference. This geospatial reference may be explicit or implicit. Explicit references include geographic coordinates measured by a smartphone's built-in location capabilities, for example by accessing a Global Navigation Satellite System (GNSS) sensor or nearby Wi-Fi access points. Alternatively, an explicit place tag can be added by the user. Implicit references include a place or location name that a user mentions in their post. Most geo-social media analysis approaches use explicitly georeferenced data with a GNSS reference, because of their high accuracy, technical accessibility and lack of ambiguity. However, only a small share of social media posts (roughly 2–10%) are explicitly georeferenced (Cheng et al., 2010; Laylavi et al., 2016). The low number of georeferenced posts reduces the sample size used for analysis, thereby compromising the reliability of the results.

Although some research efforts have been made over recent years to identify precise user locations in social media posts, challenges still remain in extracting and geocoding places with high recall and fine spatial granularity. This paper addresses these shortcomings through a customized workflow for inferred place extraction from tweets, and compares two different geocoding methods: DBpedia Spotlight (DBpedia) and a tailored workflow using spaCy, for location extraction; OpenStreetMap (OSM) Nominatim, for geocoding locations extracted by spaCy.

2 Related Work

Although Twitter offers an extensive data source, the relatively small number of explicitly georeferenced tweets poses a challenge in validating the authenticity of a tweet text. Over the years, research has been conducted to increase the percentage of geotagged tweets by extracting and geocoding implicit locations within posted tweets. For example, Das & Purves (2019) geocoded inferred locations in tweets to detect traffic events; Yaqub et al. (2018) used geocoding of inferred locations of tweets to map user sentiments during the 2016 US presidential elections. Although both studies showed high percentages of extracted locations, their results did not include a validation of the geocoded places.

Lee et al. (2014) extracted and geocoded locations from tweet posts linked to Foursquare. Their results found that 34% of the extracted locations were within a 250 m radius of the GNSS position provided. Whilst this result seemed highly satisfactory, the increase in georeferenced locations found by Lee et al. is biased towards Foursquare users and is not representative of the population of social media users as a whole. Another closely related study was conducted by (Laylavi et al., 2016), who geocoded tweet locations by giving priority to the finest-grained locations from textual content, or from users' profile locations, or from place-labelled locations. Their approach returned an accuracy of 60% of predicted locations within a 10 km radius, surpassing most state-of-the-art extractions. However, their results were obtained from a final sample of just 2,409 tweets out of ~90k tweets. In this research, we attempt to return a high accuracy of geocoded locations with a minimal loss of tweet sample size.

3 Methodology

The primary focus of this research is on extracting and geocoding inferred locations from tweets to increase the number of georeferenced social media posts for geospatial analysis. Our study uses two established tools for named entity recognition, namely DBpedia (Mendeset al., 2011) and spaCy's pre-trained 'en_core_web_trf' model.1 We used both DBpedia and spaCy in order to better assess the performances of the two models for future studies.

DBpedia Spotlight builds upon Wikipedia data and provides links to DBpedia resources following a four-stage process of entity extraction, outlined in (Mendes et al., 2011). spaCy, on

[1] https://spacy.io/

the other hand, extracts entities based on the context of a word's use and is hence not limited to a list of entities within a gazetteer, as is the case with DBpedia. However, unlike DBpedia which can perform both extraction and geocoding, spaCy does not have a built-in geocoding service. We therefore used OSM's Nominatim to geocode entities extracted using spaCy; DBpedia performed both tasks itself. Figure 1 shows our general workflow. Detailed steps in chronological order will be found in section 3.1.

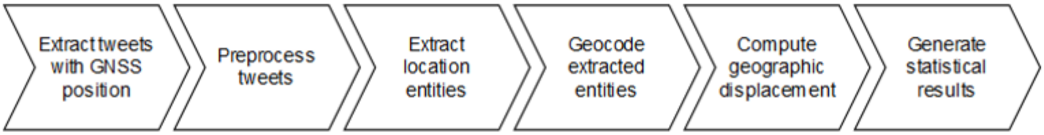

Figure 1: General Workflow.

3.1 Detailed workflow

Data: To enable the validation of our method's performance, we based our analysis exclusively on tweets containing a GNSS position; the performance is measured through the displacement distance between the geocoded location and the tweet's given GNSS position. We used a random subset of 56,052 tweets posted within the San Francisco Bay Area in California, USA, between April 2013 and May 2019. After pre-processing this dataset, we ended up with 50,616 tweets.

Pre-processing: Tweets are short, unstructured and noisy. The nature of the unstructured text makes pre-processing necessary to allow applicability of natural language processing methods. We performed text pre-processing in five stages.

First, we removed non-English tweets to simplify our analysis. Second, we substituted @ characters with the word 'at' to avoid false negatives in entity extraction due to non-matching words to be looked up in the DBpedia gazetteer, and ambiguous syntax for spaCy entity prediction. Third, we removed emojis, web addresses and hash-tag signs as we could not extract any locational information from these characters. We then discarded empty cells that resulted from the preceding step. Since our analysis was greatly interested in extracting the user's current location, as a final pre-processing step, using keyword filtering, we eliminated tweets with either future or past locational reference, and automatically-generated tweets such as weather, news or marketing messages.

Location extraction: DBpedia's performance in entity extraction is based on values assigned to the parameters 'confidence' (range between 0 and 1) and 'support' (integer values starting from 1) (Mendes et al., 2011). While high-confidence values increase accuracy, they risk omitting valuable entities. Likewise, higher support values discard all entities with Wikipedia in-links of less than the defined support value. Since we did not have an annotated dataset, we manually checked 100 tweets for the best support and confidence value pairs. A confidence level of 0.4 with a support value of 10 gave a more acceptable balance for both accuracy and recall.

The spaCy pre-trained model has locational entities divided into four classes: Geopolitical Entities (GPE), Facilities (FAC), Organisations (ORG) and Locations (LOC). GPEs are administrative units such as countries, states and cities. FACs include buildings, airports, highways, bridges etc., while the class ORG includes companies, agencies and institutions. LOC defines remaining location entities like street names, mountains, lakes or rivers. Using spaCy's pre-trained 'en_core_web_trf' model, we first extracted all four types of location entity separately. We then paired LOC, FAC and ORG to the corresponding GPE entity (when available) in order to reduce locational ambiguity between common entities such as building names.

Displacement computations: To assess the geocoding accuracy, we computed the displacements as geodesic distances between Twitter's GNSS points and the coordinates that resulted from using either DBpedia or Nominatim.

Generating statistical results: We computed the frequency distributions of displacement values for DBpedia and spaCy. We then evaluated various groupings of spaCy entities to find an entity grouping that gave higher accuracy and recall in predicting the user's location. We gave higher priority to precise locations (i.e. FAC, ORG and LOC linked to a corresponding GPE entity) than to individual location entities such as ORG or LOC. It should be noted that a geocoded tweet was counted only once in the grouping regardless of possibly having more than one geocoded entity. That is, a tweet with FAC and GPE was counted once in the group of FAC_GPE and discarded from both FAC and GPE individually.

4 Results

Figure 2 presents an overview of the number of tweets with an extracted location, and the percentage of geocoded locations. Overall, spaCy returns a higher number of entities (18,448) in comparison to DBpedia (11,701). However, DBpedia shows a higher percentage (96.0%) of geocoded entities than Nominatim (91.8%) for combined spaCy entities. When analysing spaCy entities separately, our results show lower geocoded percentages, especially for the precise locations. LOC_GPE returned approximately 68.0% geocoded locations, while ORG_GPE and FAC_GPE returned fewer than 50% geocoded locations. The FAC and ORG entities also showed relatively lower percentages of geocoded locations (70.9% and 58.9% respectively).

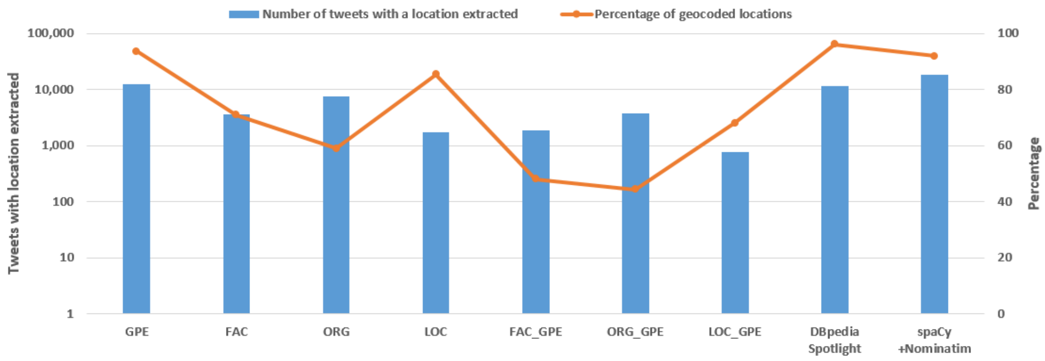

Figure 2: Number of tweets with location entity extracted (log scaled); and percentage of geocoded entities for DBpedia, and spaCy with Nominatim.

The statistical results of the computed displacements for spaCy's geocoded entities are shown in Table 1. Overall, the precise locations, FAC_GPE, ORG_GPE and LOC_GPE, show higher cumulative percentages for lower displacements (81.9%–87.4% within 10 km radius) in comparison to the individual location entities (28.2%–74.2% within a 10 km radius). On the other hand, the individual locations returned higher absolute numbers (1,493–11,426) compared to the precise locations (530–1,676).

Table 1: Displacement statistics for spaCy geocoded locations.

Cumulative frequency					
Displacement	1km	5km	10km	50km	>50km
GPE	2,816	7,209	8,482	9,437	11,426
FAC	911	1,085	1,118	1,167	2,535
ORG	1,041	1,203	1,250	1,451	4,431
LOC	485	609	666	777	1,493
FAC_GPE	601	737	767	817	878
ORG_GPE	940	1,246	1,393	1,481	1,676
LOC_GPE	318	411	434	469	530

Cumulative percentage					
Displacements	1km	5km	10km	50km	>50km
GPE	24.6	63.1	74.2	82.6	100.0
FAC	35.9	42.8	44.1	46.0	100.0
ORG	23.5	27.1	28.2	32.7	100.0
LOC	32.5	40.8	44.6	52.0	100.0
FAC_GPE	68.5	83.9	87.4	93.1	100.0
ORG_GPE	56.1	74.3	83.1	88.4	100.0
LOC_GPE	60.0	77.5	81.9	88.5	100.0

spaCy geocoded entities were grouped starting with precise locations (Group A), with further groups being created as follows:

Group A = FAC_GPE + LOC_GPE + ORG_GPE.
Group B = Group A + GPE.
Group C = Group B + FAC.
Group D = Group C+ LOC.
Group E = Group D + ORG.

Priority was given to entities with the highest cumulative percentage within a 5 km radius (see Table 1). Table 2 shows the displacement results of the DBpedia and spaCy entity groupings: there is an inverse relationship between the cumulative frequency and percentage of each displacement class. DBpedia showed a lower overall cumulative percentage within a 5 km

displacement compared to spaCy's groupings, and the lowest cumulative frequency of geocoded entities apart from Group A.

Table 2: Displacement statistics for groupings of spaCy geocoded locations and DBpedia

Cumulative frequency					
Displacement	1km	5km	10km	50km	>50km
Group A	1,823	2,343	2,541	2,712	3,017
Group B	4,401	8,752	10,021	11,078	13,108
Group C	4,866	9,301	10,583	11,662	14,373
Group D	5,076	9,584	10,902	12,044	15,171
Group E	5,516	10,081	11,417	12,610	16,936
DBpedia	3,294	6,172	7,947	9,012	11,235

Cumulative percentage					
Displacement	1km	5km	10km	50km	>50km
Group A	60.4	77.7	84.2	89.9	100.0
Group B	33.6	66.8	76.4	84.5	100.0
Group C	33.9	64.7	73.6	81.1	100.0
Group D	33.5	63.2	71.9	79.4	100.0
Group E	32.6	59.5	67.4	74.5	100.0
DBpedia	29.3	54.9	70.7	80.2	100.0

5 Discussion and Conclusion

This study set out with the aim of proposing a workflow for extracting locational information from tweets. Using tweets with GNSS positions, we extracted and geocoded location mentions in tweet texts and compared these locations with the tweets' GNSS positions. We presented two sets of results: (1) locations extracted and geocoded by DBpedia Spotlight; (2) locations extracted by spaCy and geocoded using Nominatim.

We note that our location extraction and geocoding methods will have great potential to yield finer results, once a more detailed and comprehensive analysis has been carried out. Our groupings using spaCy (Table 2) showed remarkable results, with 84.2% being within a 10 km radius of the actual user location. This result surpasses that of (Laylavi et al., 2016), who returned only 60% for the same radius and sample size of tweets. Although these results seemed satisfactory, the high percentages were achieved at the expense of sample size. Hence, we present as our main results spaCy's Group B, which retained a high sample size (10,021), with 76.4% of the retained tweet locations within a 10 km radius. DBpedia retained a rather lower sample size (7,924), with 70.7% within a 10 km radius.

Multiple reasons help explain our failure to obtain 100% accuracy. First, although keyword filtering was used to remove location mentions that were not the user's actual location, we admit that this method was not robust. Undesired tweets were likely to have been left in the sample due to mismatches or ambiguous sentence structures leading to errors in geocoding the users' actual locations. Second, the presence of ambiguous place names leads to obtaining wrong locations. Organisation entities such as 'McDonalds' were all geocoded to the headquarters, leading to large displacement values. When combined with a GPE entity, the displacement was reduced but also geocoded to the main store/location, despite there being potentially multiple locations in one city.

Our next set of steps for a complete analysis will hence be to increase the grammatical filtering of place mentions, and to combine tweets from single users to disambiguate mentioned locations based on locational trend analysis. In terms of geocoding with Nominatim, our results have shown that although Nominatim is well capable of geocoding locations down to street level, it fails to geocode place names written in a different syntax. This concurs with the

finding of (Di Rocco et al., 2016). We take note of this limitation and make two proposals for a more comprehensive assessment. First, we consider additional pre-processing steps before geocoding extracted locations. Second, we propose linking Nominatim OSM to other geocoding services.

References

Cheng, Z., Caverlee, J., & Lee, K. (2010). You are where you tweet: A content-based approach to geo-locating Twitter users. International Conference on Information and Knowledge Management, Proceedings, June, 759–768. https://doi.org/10.1145/1871437.1871535

Das, R. D., & Purves, R. S. (2019). Exploring the Potential of Twitter to Understand Traffic Events and Their Locations in Greater Mumbai, India. IEEE Transactions on Intelligent Transportation Systems, 21(12), 5213–5222.

Di Rocco, L., Bertolotto, M., Catania, B., Guerrini, G., & Cosso, T. (2016). Extracting fine-grained implicit georeferencing information from microblogs exploiting crowdsourced gazetteers and social interactions. AGILE International Conference on Geographic Information Science.

Laylavi, F., Rajabifard, A., & Kalantari, M. (2016). A multi-element approach to location inference of Twitter: A case for emergency response. ISPRS International Journal of Geo-Information, 5(5), 1–16. https://doi.org/10.3390/ijgi5050056

Lee, K., Ganti, R. K., Srivatsa, M., & Liu, L. (2014). When twitter meets foursquare: Tweet location prediction using foursquare. MobiQuitous 2014 - 11th International Conference on Mobile and Ubiquitous Systems: Computing, Networking and Services, 198–207. https://doi.org/10.4108/icst.mobiquitous.2014.258092

Mendes, P. N., Jakob, M., García-Silva, A., & Bizer, C. (2011). DBpedia spotlight: shedding light on the web of documents. Proceedings of the 7th International Conference on Semantic Systems, 1–8.

Yaqub, U., Sharma, N., Pabreja, R., Chun, S. A., Atluri, V., & Vaidya, J. (2018). Analysis and visualization of subjectivity and polarity of Twitter location data. Proceedings of the 19th Annual International Conference on Digital Government Research: Governance in the Data Age, 1–10.

Yu, M., Bambacus, M., Cervone, G., Clarke, K., Duffy, D., Huang, Q., Li, J., Li, W., Li, Z., Liu, Q., Resch, B., Yang, J., & Yang, C. (2020). Spatiotemporal event detection: a review. International Journal of Digital Earth, 13(12), 1339–1365. https://doi.org/10.1080/17538947.2020.1738569

View publication stats
