# Supplementary material for: Enhanced geocoding precision for location inference of tweet text using spaCy, Nominatim and Google Maps. A comparative analysis of the influence of data selection
Source: PLoS One. 2023 Mar 15;18(3):e0282942. doi: 10.1371/journal.pone.0282942 (PMC10016707; doi:10.1371/journal.pone.0282942)
Supplement: S1 Appendix — (DOCX) [file pone.0282942.s002.docx]

# Appendix

Keyword phrases:

1. Historical location references

'arrived from |had gone to |coming from |I was in |we were in |I was at | was at | was in | were at | went to |landed from |passed through| had visited | had gone to| flew from |flew in from| back from | miss being in | was leaving in | was staying at | was leaving at | was staying in | im from | am from | are from |moved from| originally from | grew up in |

1. Future location references

driving to| Taking the train to| taking the car to| taking the bus to| heading to| headed for| leave for| leaving for | will go to | travel to |trip to| travelling to | moving to| relocating to | flying to | will be going to| will be at | will be in | next stop | move to | transferring to | off to |departing for |on my way to| on our way to'
