# Supplementary material for: Enhanced geocoding precision for location inference of tweet text using spaCy, Nominatim and Google Maps. A comparative analysis of the influence of data selection
Source: PLoS One. 2023 Mar 15;18(3):e0282942. doi: 10.1371/journal.pone.0282942 (PMC10016707; doi:10.1371/journal.pone.0282942)
Supplement: Attachment — Submitted filename: Response to Reviewers.docx [file pone.0282942.s003.docx]

**Rebuttal letter**

PONE-D-21-27579

High precision for location extraction for social media posts using spaCy, Nominatim and Google Maps API.

PLOS ONE

Thank you for the opportunity to address the comments from the Reviewers and the Committee. The authors hope that the Reviewers and Editors will be satisfied with the amendments which we have made to the manuscript after taking into account the feedback provided.

**Report from PLOS ONE**

Thank you for submitting your manuscript to PLOS ONE. After careful consideration, we feel that it has merit but does not fully meet PLOS ONE’s publication criteria as it currently stands. Therefore, we invite you to submit a revised version of the manuscript
that fully addresses the points raised by the two reviewers.

When submitting your revision, we need you to address these additional requirements.

1. Please ensure that your manuscript meets PLOS ONE's style requirements, including those for file naming. The PLOS ONE style templates can be found at

https://journals.plos.org/plosone/s/file?id=wjVg/PLOSOne_formatting_sample_main_body.pdf and

<https://journals.plos.org/plosone/s/file?id=ba62/PLOSOne_formatting_sample_title_authors_affiliations.pdf>"

Response: The authors have ensured that the manuscript meets the PLOS ONE’s style requirements.

2. We note that you have stated that you will provide repository information for your data at acceptance. Should your manuscript be accepted for publication, we will hold it until you provide the relevant accession numbers or DOIs necessary to access your data. If you wish to make changes to your Data Availability statement, please describe these changes in your cover letter and we will update your Data Availability statement to reflect the information you provide.

Response: The authors will make all data available upon acceptance.

3. We note that [Figure 3] in your submission contain [map/satellite] images which may be copyrighted. All PLOS content is published under the Creative Commons Attribution License (CC BY 4.0), which means that the manuscript, images, and Supporting Information files will be freely available online, and any third party is permitted to access, download, copy, distribute, and use these materials in any way, even commercially, with proper attribution. For these reasons, we cannot publish previously copyrighted maps or satellite images created using proprietary data, such as Google software (Google Maps, Street View, and Earth). For more information, see our copyright guidelines: http://journals.plos.org/plosone/s/licenses-and-copyright.

We require you to either (1) present written permission from the copyright holder to publish these figures specifically under the CC BY 4.0 license, or (2) remove the figures from your submission:

a. You may seek permission from the original copyright holder of Figure 3 to publish the content specifically under the CC BY 4.0 license.

We recommend that you contact the original copyright holder with the Content Permission Form (http://journals.plos.org/plosone/s/file?id=7c09/content-permission-form.pdf) and the following text:

“I request permission for the open-access journal PLOS ONE to publish XXX under the Creative Commons Attribution License (CCAL) CC BY 4.0 (http://creativecommons.org/licenses/by/4.0/). Please be aware that this license allows unrestricted use and distribution, even commercially, by third parties. Please reply and provide explicit written permission to publish XXX under a CC BY license and complete the attached form.”

 Please upload the completed Content Permission Form or other proof of granted permissions as an ""Other"" file with your submission.

 In the figure caption of the copyrighted figure, please include the following text: “Reprinted from [ref] under a CC BY license, with permission from [name of publisher], original copyright [original copyright year].”

b. If you are unable to obtain permission from the original copyright holder to publish these figures under the CC BY 4.0 license or if the copyright holder’s requirements are incompatible with the CC BY 4.0 license, please either i) remove the figure or ii) supply a replacement figure that complies with the CC BY 4.0 license. Please check copyright information on all replacement figures and update the figure caption with source information. If applicable, please specify in the figure caption text when a figure is similar but not identical to the original image and is therefore for illustrative purposes only.

The following resources for replacing copyrighted map figures may be helpful:

USGS National Map Viewer (public domain): http://viewer.nationalmap.gov/viewer/

The Gateway to Astronaut Photography of Earth (public domain): http://eol.jsc.nasa.gov/sseop/clickmap/

Maps at the CIA (public domain): https://www.cia.gov/library/publications/the-world-factbook/index.html and https://www.cia.gov/library/publications/cia-maps-publications/index.html

NASA Earth Observatory (public domain): http://earthobservatory.nasa.gov/

Landsat: http://landsat.visibleearth.nasa.gov/

USGS EROS (Earth Resources Observatory and Science (EROS) Center) (public domain): http://eros.usgs.gov/#

Natural Earth (public domain): <http://www.naturalearthdata.com/>

Response: The authors have removed [Figure 3] and supplied a replacement figure which is now [Figure 2] that complies with the CC BY 4.0 licence. The caption has also been modified in line with the new figure.

4. Please include a copy of Table 4.

Response: The authors have made sure to separate tables and figures likewise.

[Note: HTML markup is below. Please do not edit.]

Reviewers' comments:

Reviewer's Responses to Questions

**Comments to the Author**

1. Is the manuscript technically sound, and do the data support the conclusions?

The manuscript must describe a technically sound piece of scientific research with data that supports the conclusions. Experiments must have been conducted rigorously, with appropriate controls, replication, and sample sizes. The conclusions must be drawn appropriately based on the data presented.

Reviewer #1: Partly

Reviewer #2: Yes

2. Has the statistical analysis been performed appropriately and rigorously?

Reviewer #1: Yes

Reviewer #2: Yes

3. Have the authors made all data underlying the findings in their manuscript fully available?

The [PLOS Data policy](http://www.plosone.org/static/policies.action#sharing) requires authors to make all data underlying the findings described in their manuscript fully available without restriction, with rare exception (please refer to the Data Availability Statement in the manuscript PDF file). The data should be provided as part of the manuscript or its supporting information, or deposited to a public repository. For example, in addition to summary statistics, the data points behind means, medians and variance measures should be available. If there are restrictions on publicly sharing data—e.g. participant privacy or use of data from a third party—those must be specified.

Reviewer #1: No

Reviewer #2: Yes

4. Is the manuscript presented in an intelligible fashion and written in standard English?

PLOS ONE does not copyedit accepted manuscripts, so the language in submitted articles must be clear, correct, and unambiguous. Any typographical or grammatical errors should be corrected at revision, so please note any specific errors here.

Reviewer #1: Yes

Reviewer #2: Yes

5. Review Comments to the Author

Please use the space provided to explain your answers to the questions above. You may also include additional comments for the author, including concerns about dual publication, research ethics, or publication ethics. (Please upload your review as an attachment if it exceeds 20,000 characters)

Reviewer #1: The manuscript describes a methodology to geocode Twitter status messages’ point of origin, using the inferred location in the message textual content extracted using spaCy, and geocoded using a combination of Nominatim and Google Maps API. After the data collection, the workflow described consists on six distinct steps: 1) random sampling, 2) pre-processing, 3) extraction of location entities, 4) geocoding, 5) estimation of the displacement compared to the GNSS position “ground truth”, and 6) statistical analysis of the results within 3 radius thresholds.
The topic is current and can be of interest for the readers. The text is well structured and the references are adequate. The methodology is correctly described, and the results are presented adequately. The major limitations are also explained in the discussion. The conclusion would require more work as it reads more like an extended version of the abstract, although some paragraphs in section 5 could be considered part of the conclusion.
I have included the comments divided in three sections, 1) major issues, 2) minor issues, and 3) formatting/spelling errors.

Response: The authors thank the Reviewer for the positive feedback. We have addressed the raised points as outlined in the paragraphs below. Owing to some of the major issues, the authors made some drastic changes to address the raised issues.

MAJOR ISSUES
I think the use of “high precision” in title is misleading, because it is subjective as “high” precision/accuracy has different meanings depending on the context. I would suggest “(improved/enhanced) precision” as the method discussed in the manuscript claims better accuracy than the state of the art used as benchmark. Another aspect in the title that could be made more precise is substituting “social media” by “twitter” as no other social media service was analysed.

Response: The authors thank the Reviewer for pointing the discrepancy between the paper and the title. Following other major issues outlined below, the authors have changed the title of the paper to:

Enhanced geocoding precision for location inference of tweet text using spaCy, Nominatim and Google Maps. A comparative analysis of the influence of data selection.

The explicit georeferenced data used as the “ground truth” in the accuracy assessment is oftentimes not accurate. Beyond limitations in the location accuracy of the mobile devices themselves (especially in dense urban areas) for automatically geotagged social media posts, some metadata consists in approximate coordinates (e.g. the centroid of an administrative division), or relies on unreliable user input. I think that it is necessary that the manuscript discusses these limitations as they have the potential to significantly alter the results.

Response: Thank you for your observation. The authors agree that georeferenced tweet data is often times not accurate and may be given at higher scales leading to location estimates and not the actual position from which a post originated. However, the authors would like to point out that the study used only tweets with a precise GNSS position (Latitude and Longitude coordinate pairs) and not a place tag. Thus the accuracy assessment was based on the most precise data. The authors have adjusted the explanation in the manuscript to provide more clarification of the accuracy of the ground truth data, taking into account the raised concern.

The reason for the random selection mentioned on paragraph 182-184 is explained as being chosen for “computational reasons”. I think that the reason because it was not feasible to process the complete set of 5.6 million tweets should be clarified, in particular whether it was impossible or it was a practical decision, as well as the justification of the chosen sample size.

Response: Thank you for identifying this lack of clarity. The authors revised the method section including the data selection procedure and have provided clarity to the data selection.

In addition to the decision to work with a subset of the data, I think that is important to justify why was not stratified sampling chosen instead, weighted on the proportion of messages from each source regardless of whether they had location data or not. The explained approach makes Instagram sources overrepresented, possibly leading to overfit, which can severely impact the performance if the whole dataset was considered, which is the stated objective of the authors. In addition, another issue that should be considered is that the demographics of Instagram and Twitter are not aligned and therefore one may not be representative of another.

Response: The authors thank the reviewer for pointing out this important and sensitive research aspect. According to this comment, the authors have revised the entire paper, re-run all experiments, and have investigated the influences of having different data sources when developing location inference methods. More specifically, the authors checked the performance of the developed location inference method on a proportionate sample of a geocoded dataset (having a higher percentage of Instagram tweets) and modelled representation of non-geocoded (having a higher percentage of tweets generated from native Twitter applications such as Twitter for iPhone. From their analysis, the authors have found that choice of data selection used has high influence on the performance of a location inference models.

The minor issues identified follow in the next section. However, I would like to point out that while they are minor issues, they are chained from one to the next in a long process of manual cleaning and feature engineering steps, and their combination could lead to problems with overfitting and make the results challenging to replicate, especially since the described approach does not include separate training and test sets. This should be investigated further and mentioned in the discussion.

Response: Thank you for your pointing this out. The authors have used two separate datasets. The first dataset consists of a proportionate sample of twitter source strata from a geocoded dataset. The second dataset consists of a strategically selected dataset that models the twitter source ratios in a non-geocoded dataset.

MINOR ISSUES
I would suggest that keywords would not repeat worlds already in the title (spaCy, Nominatim, Google Maps API).

Response: The authors agree on the unnecessary repetition between the keywords and the title. The authors have made sure that the keywords used do not repeat with words in the new title.

In lines 13-14 (“With a fraction of less than 10% geotagged posts, over 90% of posts are not analysed”) and lines 35-36 (“This means that more than 90% of geosocial media posts cannot be used for analysis”) the authors should clarify that it refers to spatial analysis only, as other content (semantics, multimedia content, network connections…) can be analysed without location data. The percentage is aligned with the results of this reviewer’s findings, but should include a reference as not all readers will be familiar with this imbalance.

Response: The authors agree and have explicitly stated that the limitation is only with spatial analysis. The authors have inserted the citations that go along with the given statement.

The research uses the Nominatim and Google geocoders, but I think an explanation of whether other geocoders were also considered and why they were discarded should be included.

Response: Thank you for pointing this out. The authors have provided a reason to the use of only these two geocoder in lines 308 – 309.

The order in which the steps described in subsection 3.2 are conducted can affect the outcome. In paragraph 165-173, it would be interesting to see a number of the distribution of tweets per source, sorted in descending order.

Response: The authors have added Table 1 which shows the number of Tweets per source sorted by descending order of the total tweets and the corresponding number of selected posts per source for the two datasets used.

Alternatively, figure 3 (left) could be presented as a chart.

Response: The authors have removed the mentioned Figure 3 (left) and has presented the values as a column in Table 1.

In figure 2, the number of remaining tweets after each step should be included, or the number of discarded ones, perhaps graphically.

Response: The authors have revised Figure 2 and added the number of discarded tweets, remaining posts and the remaining sources for each stage.

It would also be interesting to know the 2 additional discarded sources in paragraph 174-181.

Response: The authors reformulated the entire data selection part and the selection of the tweet sources have been clarified.

For easier understanding, figure 3 should be split into two, and the map placed in a separate figure as both are very loosely related. If they are kept together, the caption should refer to both.

Response: Thank you. The authors agree with your suggestion and have split figure 3 (left) to Table 1 showing the distribution of tweets per post and Figure 3 (right) to Figure 2 showing the distribution of the geotagged posts within the bounding box.

The number or proportion of messages translated (line 197, lines 406-410) should be stated.

Response: Thank you. The authors agree that the proportion of translated tweets need to be specified. For the new analysis however, the authors decided to work with only English tweets to avoid introducing unnecessary bias as a result of translation errors hence no translation was done.

Likewise, in the case of messages removed by keyword filtering and temporal information matching (section 3.4.2), the resulting number of samples after both processes are explained in line 240, but not individually (keyword, temporal). The numbers could also be included in figure 5.

Response: Thank you. The authors have added the number of tweets at each stage of the preprocessing stage in Fig 3.

I am not familiar enough with Nominatim, but the Google geocoder often returns multiple locations to a query, and I assume that the first one was the only considered (line 441). However, the text mentions “most probable”, which should be clarified (is it the most precise? e.g. “ROOFTOP”).

Response: Indeed, Google Maps returns multiple locations to a single query. The authors had used the term most probable to indicate the first location returned by Google Maps as the documentation states that Google Maps returns the most popular location as the first result. However, the authors acknowledge the ambiguity in the statement and have dropped the phrase most probable.

It is also possible that Google results are biased towards returning SOME result (for commercial reasons) and therefore provide a result even for very ambiguous queries (lines 435-438).

In section 3.6 it would also be interesting to measure whether it exists a consensus in both geocoders’ results.

Response: Thank you. The authors agree with the raised point however the authors did not apply this suggestion as it was out of the study scope.

From the text it is not clear the grouping explanation in section 3.8, I think it could be easier to understand with an additional column with the corresponding grouping letter(s) in table 1.

Response: Thank you for the comment. The authors changed the Groupings to only four Groups in the new analysis. Additionally, the authors clarified the formulation of the groups.

I think it would also be necessary to provide an explanation on why were not tested individually, or on the contrary, reduce the number of groups.

Response: Thank you. The authors have reduced the number of groups to four.

Figure 6 should be sorted according to the number of entities extracted, and table 1 could follow this order for easier comparison. For clarity it would also be possible to sort only within the 1/2/3 entity groups separately.

Response: Thank you for the comment. The authors have made sure that all figures and tables are sorted in a uniform manner.

Although charts with dual axes (in this case vertical) are not advisable in general, it seems to work fine in this case to summarize the results, although the data corresponding to the second axis could appear above or below in a subpanel with its own vertical scale, sharing the horizontal scale.

Response: Thank you for the comment. We have replaced the figure completely with table 4.

In addition, I think that the use of a line chart instead of points/bars suggest a relationship which is not suitable for the data at hand, and reads like a time series.

Response: The authors agree with the reviewer’s observation. The line chart has been removed and the values previously represented as a line chart have been tabulated under table 4.

TYPOS (not exhaustive)
References
• Consolidate multiple references, e.g. [7], [8] and [9] to [7, 8, 9] in line 52, or place after each item in lines 53-54 if applicable.

Response: The references have been consolidated.

• Extra [10] reference at the end of line 55

Response: Thank you. This has been corrected.

• “Orphan” references without subject, such as [11] in line 59, [14] in line 71, [17] in line 85, [18] in line 89, [6] in line 96… (in APA style it would be acceptable)

Response: Thank you for pointing this out. The authors have adjusted the text to remove all Orphan references.

• Unnamed references, e.g. “done by [12]” in line 63, [13] in line 65…

Response: Thank you. The authors are not clear what the unnamed references are referring to in this part. If you refer to the insertion of the reference at the end of the sentence, the authors have tried to include references in text.

Missing references for some claims
• Number of tweets with location data (line 34-35)

Response: Thank you. The authors have included all references which were missing.

• “good performance” of spaCy on twitter data (line 132)

Response: Thank you. The authors have corrected this.

Use of informal language
• “we see” in line 38
• “tremendous” in line 52
• “64+” language models in line 128
• “other such” in line 129
• “cells” in line 203 is Pandas-specific

Response: Thank you. The authors have corrected and revised the manuscript to reduce informal language.
Formatting
• The bold text in lines 55, 70 and 85 would seem to work better as subsections of section 2

Response: Thank you. The authors have reformulated text and have replaced the bold text as subsections.

• Reference not found in line 168

Response: Thank you. The authors have corrected this.

• “Appendix” in line 222

Response: The authors thank the reviewer. However, the authors have failed to identify the error in the outlined word.

• Reference not found in lines 403-404

Response: Thank you. The authors have corrected this.

Misspellings
• “profiled” in line 59

• Lower case Google in line 61

• Lower case python in line 127

• “\” in line 156

Response: The authors have addressed the comments above and have revised the manuscript in check for additional misspellings.

Reviewer #2: The study aims to geocode the non-geotagged social media content with a proposed methodology. I believe the study should include the geo-parsing studies (geoparsepy, geoparser.io, unlock etc.) in the introduction part to provide wide perspectives of how spaCy works.

Response: Thank you. The authors discussed location inference methods developed on different geoparsers in the related work section. Additionally the authors made a comparison on the different geoparsers with spaCy in the discussion section.

In the geocoding part the proposed 4 approach should be exemplified step by step. How the Nominatim + Google Maps API and Google Maps API on Nominatim geocoded locations impact the results?

Response: Thank you for the comment. The authors have reduced the approaches to only Nominatim and Google Maps API in line with the new analysis made.

For the displacement granularity (1, 10, 50km), it might be good to look at the city or county area and re-determine the granularity.

Response: The authors agree that the granularities could also be defined at city or country level. However, the objective of the study was to geolocate with high precision the position of the user at the time of sending each post. For this reason, the authors felt that the displacement of up to 50km was adequate.

In the methodology part, how the data flow tackles with the duplicating place names (chain stores, coffee shops, county names) should be identified. A flow diagram would be more readable for it.

Response: Thank you. We amended the description of the issue of duplicate place names in the text – like this, it is simpler to understand without having to add another figure for this process.

Used Twitter data are generated from several platforms (Instagram, foursquare,..) and this means the attached location copied their places libraries and easily be manipulated manually. This might be good point to add this while discussing the locational accuracy of each tweet.

Response: Thank you for the response. The authors have added a new analysis and investigated the different data sources of Twitter data as such the authors have provided a discussion of the location accuracies of the returned tweets.

6. PLOS authors have the option to publish the peer review history of their article ([what does this mean?](https://journals.plos.org/plosone/s/editorial-and-peer-review-process#loc-peer-review-history)). If published, this will include your full peer review and any attached files.


If you choose “no”, your identity will remain anonymous but your review may still be made public.

**Do you want your identity to be public for this peer review?** For information about this choice, including consent withdrawal, please see our [Privacy Policy](https://www.plos.org/privacy-policy).

Reviewer #1: No

Reviewer #2: **Yes:** Ayse Giz Gulnerman

[NOTE: If reviewer comments were submitted as an attachment file, they will be attached to this email and accessible via the submission site. Please log into your account, locate the manuscript record, and check for the action link "View Attachments". If this link does not appear, there are no attachment files.]

While revising your submission, please upload your figure files to the Preflight Analysis and Conversion Engine (PACE) digital diagnostic tool, <https://pacev2.apexcovantage.com/>. PACE helps ensure that figures meet PLOS requirements. To use PACE, you must first register as a user. Registration is free. Then, login and navigate to the UPLOAD tab, where you will find detailed instructions on how to use the tool. If you encounter any issues or have any questions when using PACE, please email PLOS at [figures@plos.org](mailto:figures@plos.org). Please note that Supporting Information files do not need this step.

Response: The above listed items have been addressed in the revised manuscript. The stated references within the manuscript are shown under the Reference list.

Reference list

[1]	Huang B, Carley KM. A large-scale empirical study of geotagging behavior on twitter. Proc 2019 IEEE/ACM Int Conf Adv Soc Networks Anal Mining, ASONAM 2019 2019:365–73. https://doi.org/10.1145/3341161.3342870.
