# Supplementary material for: Enhanced geocoding precision for location inference of tweet text using spaCy, Nominatim and Google Maps. A comparative analysis of the influence of data selection
Source: PLoS One. 2023 Mar 15;18(3):e0282942. doi: 10.1371/journal.pone.0282942 (PMC10016707; doi:10.1371/journal.pone.0282942)
Supplement: Attachment — Submitted filename: Response to Reviewers.docx [file pone.0282942.s004.docx]

Rebuttal letter

Response: The authors would like to thank the Academic editors and the Reviewers for the opportunity to address the comments. The authors hope that the Editors and Reviewers will be satisfied with the amendments which we have made to the manuscript after taking into account the feedback provided.

**Reviewer #1**: The authors have adequately addressed the comments mentioned in the first review round and the manuscript and this reviewer feels that it is now acceptable for publication after correcting the minor issues listed below. In addition, the paper has been revised to include the analysis of the influence of having different data sources, for example Instagram, and have included a discussion in section 5.1. In the case of Instagram, the reviewer would like to point out that the location is oftentimes introduced by the users themselves manually in a map, instead of using GNSS, which could arguably explain the differences in performance.

Response: The authors thank the reviewer for the positive feedback. The authors agree with the reviewer that Instagram does not always rely on GNSS for location sharing. The last paragraph of the discussion section points out to the reviewers point.

There are some identified minor issues (not exhaustive)

- GNSS should be defined elsewhere than the abstract on first mention.

Response: Thank you: The authors have removed the GNSS from the abstract and defined it on line 148 which is its first appearance in the text.

- "Error! Reference source not found" in lines 169, 178, 179, 180, 181, 192, 212, 222, 257, 290, 308, 312, 316, 364, 380, 446

Response: The authors thank the reviewer for pointing this out. The references must have been altered during saving of the document. The authors have corrected all instances where cross reference is not found and hope that the cross reference will not have additional issues.

- Decimal comma instead of thousands separator in line 347

Response: Thank you. The authors have fixed the document and cross-checked for any other similar instances.

- Table 7 is shown almost a full page after being referred in line 398

Response: The authors thank the reviewer for pointing this out and have moved Table directly below its first mention.

Duplicated section 5.1 in line 466 that should be 5.2

Response: Thank you. The authors have corrected the duplicate.

As a final suggestion, it would be interesting to know if the recent changes in Twitter ownership could impact the applicability of the findings in the future.

Response: The authors thank the reviewer for bringing this up. Yes, indeed there has been a lot of speculation on foreseeable impacts of most Twitter analysis. In terms of future applicability of location inference models, the authors do not foresee any major impact. In a probably worst case scenario where real time tweets are not publicly available or a huge number of people uninstall Twitter, location inference models can always be applied to historical dataset or adopted to match different social media datasets.

**Reviewer #3**: The paper presents a method to geolocate tweets based on their textual content. Actually, location inference is at the core of the subject, aiming to get as much geocoding precision as possible from references to places made in the (usually terse) tweet text. Validation is achieved using a fully geotagged tweet dataset, in which geographic coordinates are associated to each message.

Being this a first review on a second-version paper, I did not find much cause for major revisions. Methodologically the paper is sound, used a good ground-truth-based validation process, and presents its findings accurately and in sufficient detail. Data are available online at Harvard’s repository.

The paper’s subject has been intensively explored in years past, especially in the early 2010s, a time in which most people did not care or were unaware of privacy concerns and allowed Twitter to release the geolocation at which a tweet was issued. This provided researchers with lots of geotagged messages to explore, and therefore several interesting and important articles can be found from around that time. However, this set of contributions is left out of the literature review, which concentrates on more recent work. It’s a pity that there has been no connection with works from a time in which Twitter provided much more ground truth data than today, when users in general have blocked their location to be associated with tweets.

There are even interesting works from 10 or 15 years ago, or even earlier, that revolve around the so-called “spatial prepositions”, expressions that are used in text in relation to toponyms and other references to places. In a sense, this is what the present paper proposes, including the comparison of toponym candidates against a reference dataset (some gazetteer back then; OSM, spaCy and Google now). The idea of single, double, triple references in a single message has also been used successfully in the past, trying to geolocate traffic events from Twitter.

Nevertheless, at the time of these contributions, data was not usually provided for replicability purposes, so the authors could only have access to ideas and techniques that could be compared to their proposals.

In conclusion, the paper is sound, is very well written and organized, uses a good and publicly available dataset with adequate annotations with which to perform analyses and comparisons. The observation in the conclusions that the geocoder has a significant influence in the results comes as no surprise, since this has been reported in several of the works I mentioned earlier. It also must be said that some of the ideas and techniques that are employed in the paper have been tried before, including (1) extraction of location indications from the tweet content, (2) analysis of expressions that indicate a location (spatial prepositions), (3) use of gazetteers and addressing databases (not necessarily embedded in a geocoding product), (4) improved reliability of messages in which multiple references appear, (5) using past messages and user relationships in the definition of a tweet's location. I’d only wish that the authors’ literature review reached further back so that they could get to know and recognize some ideas presented earlier in geocoding, geographic information retrieval, toponym resolution, location-based social networks and other subfields.

Response: The authors thank the reviewer for the positive feedback. The authors agree with all points raised by the reviewer. We have widened our literature review to incorporate earlier studies. In light of new understanding, the authors have also refined sections of the literature review, discussion and conclusion to acknowledge existing findings and clarify the contribution of the paper to the research community.

**Reviewer #4**: - The major concern is that the contributions of this article are not clear. The authors seem to use existing data, existing methods, and existing tools.

Response: The authors thank the reviewer for the feedback. The authors agree that existing methods were used. What the authors show, however, is that by following a different methodological setup, there are able to produce higher precision on a random sample of a geotagged dataset.

- At the same time the authors claim that they have developed a "new high precision geocoding approach" - The authors should explicitly describe what their approach is and how it is different to existing approaches.

Response: Thank you for your feedback. The authors agree that the wording was misleading. As such, the authors have revised the manuscript and stated that they enhance existing methods. The authors have acknowledged the use of existing approaches and clarified their contribution in the last paragraph of the previous work section under the subsection ‘Tweet’s point of origin’

- The motivation for filtering based on apps is not clear. Sure, sampling based on data source will impact the result because different apps create different user behavior and data. But why is this relevant? And why is this relevant for this paper in which a new geocoding approach is being developed?

Response: The use of different datasets and data source distribution was done to show how location inference models trained on a proportionate distribution of a geotagged dataset may be impacted when applied to a sample of a non-geotagged dataset where the Tweet source distribution differs.

Our reason to include this analysis was based on the comments from the previous review who stressed out this possible discrepancy in model transferability. The authors found the issue of different data source distribution to be one that has been overlooked when developing location inference models and decided to include this analysis hand in hand with the proposed location inference approach. The authors believe that combining these two aids weight to the paper.
